# Controversies and Recent Advances in Senescence and Aging

**DOI:** 10.3390/cells12060902

**Published:** 2023-03-15

**Authors:** Nicole Wagner, Kay-Dietrich Wagner

**Affiliations:** CNRS, INSERM, iBV, Université Côte d’Azur, 06107 Nice, France

Aging is the leading predictive factor of many chronic diseases that account for most of the morbidity and mortality worldwide, i.e., neurodegeneration, cardiovascular, pulmonary, renal, and bone diseases, as well as cancers. Oxidative stress and reactive oxygen species generation, over-production of inflammatory cytokines, the activation of oncogenes, DNA damage, telomere shortening, and the accumulation of senescent cells are all widely accepted mechanisms contributing to aging. Senescence is mainly thought to be provoked by negative cellular stress but might also be induced by physiological developmental stimuli. Senescence is characterized by irreversible cell cycle arrest independent of quiescence and terminal differentiation. However, more recent observations suggest that the status of developmental and cancer senescent cells might not be irreversible. Aside from cell cycle arrest, senescent cells are characterized by morphological changes and molecular damage, metabolic alterations, and a specific secretory phenotype (SASP). Senescent cells contribute to embryonic development and participate in tissue repair and tumor sup-pression, but they are also involved in detrimental tissue decline during aging. Thus, the application of senolytic or senostatic drugs to halt or reverse age-related pathologies could represent an interesting therapeutic option. This Special Issue of *Cells* compiles novel and exciting insights into the mechanisms of aging and senescence.

Carrillo-Salinas and colleagues describe the effects of short-chain fatty acids on the function of neutrophils in young and older women with relevance for HIV infection risk [1]. HIV infection risk is high in younger women, but new HIV infections in older women are rising worldwide. Vaginal microbiota represent a defense against infections including HIV. Alterations in the physiological vaginal bacterial populations occur alongside other stimuli in older women as well. High concentrations of short-chain fatty acids are the result of vaginal dysbiosis. The authors compared the response of neutrophils from younger and older woman to short-chain fatty acids. In response to HIV stimulation, short-chain fatty acids reduced the chemokine secretion of neutrophils of young and older women. In addition, incubation with pathological concentrations of short-chain fatty acids diminished the activation and migration of neutrophils from older women and reduced the secretion of alpha defensins as molecules with antiviral activity. These interesting results do not only show that vaginal dysbiosis via short-chain fatty acids reduces neutrophil function but that these perturbations become more prominent with increasing age. The data also suggest that the re-establishment of physiological vaginal microbial flora with a resulting decrease in short-chain fatty acids might be a relatively simple way to reduce to some extend the risk of infections, especially in older populations.

Oxidative stress is generally assumed to increase with aging and induce senescence which might have consequences for one’s lifespan. Reducing neuronal oxidative stress is known to extend the lifespan in Drosophila [2]. However, the exact source for reactive oxygen species in this model is not fully understood. Baek and colleagues published in this Special Issue of *Cells* the identification of dual oxidase (Duox) as a source for reactive oxygen species (ROS) in Drosophila melanogaster [3]. Duox is activated by intracellular calcium to produce H_2_O_2_. Duox heterozygous male flies showed an expected reduced expression of Duox by 50%, as well as decreased ROS and H_2_O_2_ production. They survived longer under standard conditions as well when they were exposed to ROS-producing food. Whether neuroinflammation and senescence in this model are reduced remains to be determined. Nevertheless, the data nicely support the critical involvement of reactive oxygen species in lifespan determination in this model. Unfortunately, the relations between antioxidants, ROS production, and human health and longevity seem to be more complex [4,5].

Ke et al. determined ROS production in retinal pigment epithelial (RPE) cells [6]. They compared mononucleated and multinucleated RPE cells and determined that under baseline conditions, ROS production was similar in both cell types, while multinucleated cells had higher ROS production and DNA damage after irradiation. Surprisingly, in mice, the number of multinucleated cells was not age-dependent and the comparison between different species revealed that multinucleation seems to be a characteristic of nocturnal animals, while in humans and other diurnal species the fraction of multinucleated cells is low. Differences in ROS production and DNA damage were only detectable after the irradiation of mononucleated and multinucleated cells. As multinucleation was independent of the age of the mice, this represents another example of the dissociation of reactive oxygen species production, DNA damage, and age-related phenotypes.

Besides the deleterious effects of ROS, telomere shortening is considered to be a hallmark of aging [7]. Exercise is believed to have positive effects on telomere length and the associated shelterin complex proteins, while the opposite is the case for obesity. Nevertheless, shelterin genes show a very dynamic spaciotemporal expression pattern throughout the lifespan [8], and the effects of exercise on telomere length differ largely across multiple studies and have mostly been measured in peripheral blood cells. The group of researchers working alongside Markus Herrmann reported a careful study using exercised and sedentary rats fed with either a standard or a high-fat diet [9]. The rats were exercised for quite a long period of 10 months and telomere length and mRNA expression of telomerase, as well as the shelterin genes Terf-1 and Terf-2, were measured in multiple organs. A high-fat diet in the non-exercised control group induced telomere shortening and reduced mRNA expression for telomerase, Terf-1, and Terf-2 only in visceral fat, while in most organs no conclusive effects were observed in telomere length, telomerase, Terf-1, and Terf-2 expression in response to exercise or a high-fat diet. Nevertheless, it seems possible that such a difference might occur in response to training in very old age. A challenge for the future will be to establish training protocols and dietary interventions which might increase telomere length and delay aging.

Unfortunately, long telomeres and high telomerase activity might not only protect against aging but are also characteristic of cancer cells. Therefore, the inhibition of telomerase activity could represent an attractive therapeutic target for anti-tumor applications. Yan et al. screened a library of 800 natural compounds for potential inhibitors of telomerase activity [10]. They identified sanguinarine chloride as being an inhibitor of telomerase expression and activity. This compound inhibits the growth of several cancer cell lines in vitro and of xenograft tumors in vivo. The safety and efficacity as a potential drug candidate for anti-tumor therapy in humans remains to be determined in future studies.

Another important factor driving aging is cellular senescence. Senescence is characterized by the growth arrest of cells, which was first described in fibroblasts in long-term culture [11,12] and the expression of characteristic markers and secretion of a variety of diverse molecules, the so-called senescence-associated secretory phenotype (SASP) [13]. A problem with the characterization of senescent cells is that not a single highly specific marker exists to identify these cells. Thus, the International Cell Senescence Association released a consensus statement remarking that a combination of more than two typical markers is required to identify a cell as being senescent [14]. Zimmermann and colleagues carefully investigated markers of senescence in melanocytes and melanoma cells. They induced senescence in human melanocytes via the overexpression of mutant BRAFV600E and in melanoma cells via the chemotherapeutic agent etoposide [15]. Both cell types showed increased beta-galactosidase (β-Gal) activity. As this is very common but poorly understood in the field, although all cells were exposed to the senescence-inducing stimuli, only a fraction became β-Gal positive. The cell cycle inhibitor p16INK4A was induced in both models, and as a third marker for the SASP, the authors suggested CXCL2. The use of the two independent cell systems allows important conclusions to be drawn on the choice of senescence biomarkers when working with melanocytic systems, and thus the paper serves as important guidance in the field. In the future, an enormous amount of work regarding the detection of senescence in response to different stimuli and in different cell and organ systems will have to be conducted.

Although the authors describe p16INK4A as a marker for senescence and p16Ink4a-targeting models are frequently used to eliminate senescent cells (reviewed in [13]), we reported a careful analysis of p16Ink4a expression in several organs starting from embryonic development (Embryonic Day 10) until old age in mice [16]. The expression of p16 was highly dynamic in all organs in the embryonic and postnatal stages and increased dramatically in old mice, which at this time point agrees with senescence and SASP factor expression. The expression of p19 and p21 was less variable and increased to a moderate extent in old age. Interestingly, high p16Ink4a protein expression during embryonic development coincided with organ differentiation. In old mice, we observed a predominant expression of p16 mRNA and protein in liver endothelial cells versus non-endothelial cells. This is in agreement with a recent p16 ablator mouse model, which affects liver sinusoidal endothelial cells the most prominently [17]. The expression of p16Ink4a in early life was confirmed recently in a highly sensitive reporter system in fibroblasts of the lung. These p16Ink4a-positive cells surprisingly had an enhanced capacity to sense tissue inflammation and respond through their increased secretory capacity to promote epithelial regeneration [18].

In addition, we reviewed the roles of p16INK4A, p14ARF/p19ARF, and p21 in organ development and homeostasis in this Special Issue of *Cells* [19]. We analyzed the knowledge surrounding p16INK4A, p14ARF/p19ARF, and p21 in embryonic and organ development and described in detail the data reported in the literature and the different animal models targeting these senescence-associated proteins. We highlight the most recent advancements and controversial findings, which have largely contributed to a broader understanding of the senescence mechanism and the roles of p16, p19Arf, and p21 therein. Interestingly, senescent cells do not only have detrimental effects but are also required for physiological functions and are involved in tissue repair. The SASP is not a uniform set of secret factors but differs depending on p16 or p21. The beneficial effects of senescent cell removal are most likely due to a normalization of the SASP and not merely attributed to the removal of these “non-functional” cells; finally, p21-dependent senescence is not an irreversible mechanism which leads to the clearance of the p21-expressing cells via macrophages, but can be reversible [20].

Chen and Skutella propose in their review partial senescent cell reprogramming as a strategy for anti-aging therapies [21]. They suggest that partial reprogramming can produce a secretory phenotype that facilitates cellular rejuvenation. They carefully point out that only partial reprogramming is desired to avoid tumor risk and organ failure and describe approaches for achieving this goal. The authors review the strategies for reversing senescence and the potential underlying mechanisms, identify candidates for this approach, and develop clinical translational strategies to achieve partial reprogramming of senescent cells with the aim of increasing people’s healthy lifespan and reducing frailty. This review has already attracted a very broad audience.

Hong and colleagues focus in their review on the molecular mechanisms of alveolar epithelial stem cell senescence and the senescence-associated differentiation disorders in pulmonary fibrosis [22]. The topic is of high actual interest as SARS-CoV-2 viral infections induce acute pulmonary epithelial cell senescence, which is followed by fibrosis and largely determines the disease outcome. The authors focus on the TGF-β signaling pathway inducing the suppression of telomerase activity and thereby inducing senescence of the alveolar epithelial stem cell and pulmonary fibrosis. Alternatively, dysregulation of the shelterin complex protein TPP1 mediating the DNA damage response, pulmonary senescence, and fibrosis is discussed. They highlight studies indicating that the development of senescence-associated differentiation disorders is reprogrammable and reversible by inhibiting stem cell replicative senescence in pulmonary fibrosis, and provide a framework for the targeted intervention of the molecular mechanisms of alveolar stem cell senescence and pulmonary fibrosis.

Cellular senescence in aging lungs and lung diseases is reviewed in this Special Issue of *Cells* by Aghali and colleagues [23]. They provide a successful overview of cellular senescence, as well as the known signaling pathways and biomarkers of senescence. The role of cellular senescence in chronic obstructive pulmonary disease (COPD) and idiopathic pulmonary fibrosis (IPF) is reviewed in detail. Furthermore, the implications of mitochondrial alterations and mitochondrial DNA mutations in senescence and aging in the lung are discussed. Finally, the authors provide a clinically important outlook regarding senescence as a potential therapeutic target in lung diseases.

The group working with Maria Cavinato published two review articles in this Special Issue of *Cells* entitled “Controversies and Recent Advances in Senescence and Aging”. The first article deals with the topic of senescence. The authors introduce the relation between cellular senescence and skin aging and analyze in great detail the major components of air pollution on lungs and mainly skin aging. Air pollution and the consequences for senescence and aging is a highly relevant topic for countries with increased industrialization and intensified transport. Fortunately, the authors also provide guidance for tackling the consequences of air pollution on the skin by reviewing the available information on therapeutics and cosmetics in this specific field [24].

In the second article, the group summarizes the current knowledge surrounding age-related lysosomal dysfunctions [25]. Deregulated nutrient sensing, mitochondrial dysfunction, and altered intercellular communication are additional characteristics of senescent cells, which can be attributed to lysosomal dysfunction. The authors introduce lysosomal components, their structure, and lysosomal biogenetic and metabolic pathways. They describe the function of lysosomes in endocytosis, autophagy, mitophagy, and mitochondrial dysfunction, and explain in detail the lysosomal dysfunctions related to aging and senescence. As a major pathway for senescence, mTORC signaling is discussed. In terms of potential therapeutic interventions, it is interesting to note that the treatment of senescent cells with mTORC1 inhibitors ameliorates senescence phenotypes and extends the lifespan in mice [26]. In addition, increased β-galactosidase activity in the lysosomes of senescent cells might represent an opportunity to activate highly specific pro-drugs as senolytic compounds [27].

Jin and colleagues review the relation between pyroptosis, autophagy, and sarcopenia in aging [28]. Pyroptosis—cellular inflammatory necrosis—represents a form of regulated cell death, which plays a role in the ageing progress. It is closely related to age-related diseases such as cardiovascular diseases, Alzheimer’s disease, osteoarthritis, and sarcopenia. Sarcopenia refers to an aging-related loss of muscle mass. Autophagy of skeletal muscle cells can inhibit the activation of the pyroptosis pathway. The authors discuss the mechanisms of aggravated oxidative stress and poor skeletal muscle perfusion in ageing muscle, which activate the nod-like receptor (NLRP) family to trigger pyroptosis, and the role of chronic low-grade inflammation in this process.

Brauning et al. discuss natural killer cells’ phenotypes and functions in aging [29]. The age-related impairment of the immune function (immunosenescence) is one important cause of age-related morbidity and mortality. Despite an increased number of natural killer (NK) cells in aged individuals, their function is impaired with reduced cytokine secretion and decreased target cell cytotoxicity. NK cells are the central actors in the immunosurveillance of senescent cells, thus also linking the mechanisms of senescence and aging together. This excellent review describes the recent advances and open questions in understanding the interplay between systemic inflammation, senescence burden, and NK cell dysfunction in the context of aging. A profound understanding of the factors driving NK cell aging is a pre-requisite for developing potential therapies countering age-related diseases.

Last but not least, Cai and colleagues review mouse models of accelerated aging [30]. Mice are frequently used in aging and senescence research due to their similarities to humans, their short lifespan, and the ease of reproduction. Nevertheless, models of accelerated aging are highly valuable in order to decrease time and costs in aging research. This review provides excellent guidance and a description of the available models for researchers working in the field.

Taken together, the Special Issue “Controversies and Recent Advances in Senescence and Aging” comprises an excellent collection of original articles and reviews highlighting different novel aspects in the fields of senescence and aging research. They will hopefully stimulate discussions and further research in these fields which are extremely important for a constantly aging human population.
